# Association between red blood cell distribution width/albumin ratio and all-cause mortality or cardiovascular diseases mortality in patients with diabetic retinopathy: A cohort study

**DOI:** 10.1371/journal.pone.0296019

**Published:** 2023-12-21

**Authors:** Weina Fu, Feng Hu, Caiyun Xu

**Affiliations:** 1 Department of Ophthalmology, Ningbo Medical Center Lihuili Hospital, Ningbo University, Ningbo, P.R. China; 2 The Archive Room, Ningbo Medical Center Lihuili Hospital, Ningbo University, Ningbo, P.R. China; Osaka University of Pharmaceutical Sciences, JAPAN

## Abstract

**Background:**

Red blood cell distribution width/albumin ratio (RAR) has been reported as an independent risk factor for diabetic retinopathy (DR), while its association and predictive value in the prognosis of DR patients has not been reported. This study aims to explore the association and predictive value of RAR in the prognosis of DR patients.

**Methods:**

This was a retrospective cohort study based on the National Health and Nutrition Examination Survey (NHANES). The independent variable was RAR, and dependent variables were all-cause mortality and cardiovascular diseases (CVD) mortality. The association between RAR and the risk of all-cause mortality and CVD mortality was assessed using univariate and multivariate cox regression models. The results were shown as HR (hazard ratio) with 95% confidence intervals (CIs). Subgroup analysis based on age or hyperlipidemia was performed. The discrimination of the prediction model was assessed using concordance index (C-index).

**Results:**

A total of 725 eligible patients were finally included in this study. The increase of RAR was associated with increased risk of all-cause mortality (HR: 1.15, 95%CI: 1.01–1.31) and CVD mortality (HR: 1.35, 95%CI: 1.12–1.63) after adjusting the covariates. We also found the significant association between higher RAR and higher risk of CVD mortality in DR patients with age < 65 years (HR: 1.35, 95%CI: 1.09–1.67) and with hyperlipidemia (HR: 1.34, 95%CI: 1.10–1.64). C-index of RAR for all-cause mortality and CVD mortality was 0.63 (95%CI: 0.59–0.67) and 0.65 (95%CI: 0.59–0.71), respectively.

**Conclusions:**

Higher RAR was associated with the higher risk of all-cause mortality and CVD mortality in DR patients, and RAR may be a useful predictor for the prognosis of DR patients.

## Introduction

Diabetic retinopathy (DR) is one of the common microvascular complications of diabetes, which has become the main cause of irreversible blindness [1]. In 2020, the global prevalence of DR is 22.27% and it is expected to be higher by 2045 [2]. For DR patients, the risks of all-cause mortality, macrovascular events, and cardiovascular diseases (CVD) mortality significantly increase [1, 3]. Therefore, it is important to explore prognosis-related indicators for identifying DR patients with high risk of poor prognosis.

Inflammatory factors play an important role in retinal hypoxia and ischaemia, which are critical components in the development and progression of DR [4]. Red blood cell distribution width (RDW), an indicator used to evaluate the size heterogeneity of circulating erythrocyte, is traditionally used for the differential diagnosis of anemia [5]. Evidence has shown that RDW is related to inflammation, and has been identified as a novel prognostic marker that reflects chronic inflammation in diabetes patients [6]. Al-Jie et al. have reported that RDW was an independent predictor for all-cause mortality and cardiovascular complications in diabetes patients [5]. RDW combining with other viable indicators has been reported with a better discriminating ability to predict the prognosis of patients with diabetes-related complications [7].

Albumin (ALB) is an indicator synthesized in the liver, and participates in the regulation of inflammatory response [8, 9]. Wang et al. have found that serum ALB was negatively associated with the odds of DR [10]. RDW/ALB ratio (RAR) is a new combined indicator, which has been recognized as a prognostic marker reflecting chronic inflammation in patients with severe acute pancreatitis, heart failure, acute myocardial infarction, and critically ill pneumonia [11–14]. Hong et al. have found that RAR was an independent and robust marker to predict all-cause mortality in patients with diabetic foot ulcers [7]. Zhao et al. have reported RAR as an independent risk factor for DR [15]. However, the association and predictive ability of RAR in the prognosis of DR patients have not been reported.

In this study, we aimed to explore the association between RAR and the prognosis of DR patients. Also, we explored the predictive value of RAR, which may provide guidance to identify high-risk patients with poor prognosis.

## Methods

### Study design and data source

This was a retrospective cohort study based on the National Health and Nutrition Examination Survey (NHANES) (https://wwwn.cdc.gov/nchs/nhanes/Search/default.aspx). NHANES was a survey designed to assess adults’ and children’s health and nutritional status in American, and examined a representative sample of about 5,000 persons in 15 counties across the country. NHANES combined interviews and physical examinations. Interviews contained demographic characteristics, socioeconomic status, diet, and health-related questions. Physical examinations contained medical, dental, physiological, and laboratory measurements. Our study was a secondary analysis using the NHANES data from 2005–2006 and 2007–2008 because data on the retinal image were recorded in these two cycles. Ethical approval for this study was waived by the Institutional Review Board of Ningbo Medical Center Lihuili Hospital, because the data were accessed from the NHANES (a publicly available database). Written informed consent was waived by the Institutional Review Board of Ningbo Medical Center Lihuili Hospital due to retrospective nature of the study.

### Patients

Participants were included if they met the following criteria: (1) age ≥ 18 years; (2) DR diagnosed by retinal image; and (3) with RDW and ALB measurements. Exclusion criteria were as follows: (1) renal dialysis patients; and (2) cancer patients. The mean follow-up was 133.84 (2.85) months.

Diabetes was determined by the self-report of diagnosis [16]. For participants, the forty-five-degree non-mydriatic digital retinal images of each eye were captured, and each retinal image was viewed using EyeQ Lite image processing software. The presence of retinal diseases was assessed using the ophthalmic digital imaging system. DR was defined by the presence of hard exudates (HE), soft exudate (SE), intraretinal microvascular abnormalities (IRMA), venous loops (VL), microaneurysms (MAS), and hemorrhages (HEM) [17]. According to the NHANES Digital Grading Protocol, severity levels of DR were classified into no retinopathy (no retinopathy; non-diabetic retinal disease specific retinopathy; and questionable retinopathy), mild non-proliferative retinopathy (NPR) [HE, SE, IRMA, VL but no MAS; HEM only, no MAS; MAS only; and early NPR], moderate/severe NPR (moderate NPR; severe NPR), and proliferative retinopathy (PR) [fibrous proliferation only; no retinopathy but panretinal photocoagulation scars present (PRP laser); MAS only + PRP laser; early NPR + PRP laser; moderate/severe NPR + PRP laser; PR < high risk characteristics as defined in the early treatment diabetic retinopathy study (HRC); PR ≥ HRC; and total vitreous hemorrhage (VH)] [17, 18]. In our study, the severity level of DR was divided into mild group (mild NPR) and moderate/severe group (moderate/severe NPR and PR).

### Data extraction

#### Independent variable

The independent variable was RAR, which was defined as the ratio of RDW/ALB that both of them could be obtained from laboratory data files. RDW was included in the complete blood cell count (CBC) and processed by a Beckman Coulter MAXM Instrument. A single beam photometer was utilized for hemoglobinometry [19]. ALB concentration was measured using the dye bromocresol purple [20].

#### Dependent variable

Dependent variables were all-cause mortality and CVD mortality.

#### Covariates

Covariates were extracted based on baseline characteristics, physical examination, therapy, comorbidities, and laboratory parameters.

Baseline characteristics included age, gender (male and female), race, education level, marital status, poverty income ratio (PIR), duration of diabetes, metabolic equivalent (MET), smoking (smoked at least 100 cigarettes in life), drinking (at least 12 alcohol drinks one year), family history of diabetes, and family history of heart attack.

Physical examination included body mass index (BMI), which was calculated as weight (kg)/height (m)^2^.

Therapy included anti-diabetic agent, which defined as the use of insulin or oral antihyperglycemic tablets.

Comorbidities included glaucoma, macular degeneration, hypertension, CVD, depression, hyperlipidemia, chronic kidney disease (CKD), chronic liver disease (CLD), and anemia. CKD was defined as urinary albumin to creatinine ratio (UACR) > 30 mg/g and/or estimated glomerular filtration rate (eGFR) < 60 mL/min/1.73 m^2^ based on 2021 Kidney Disease: Improving Global Outcomes (KDIGO) guideline [21]. Anemia was defined as hemoglobin level < 12 g/dL in women or < 13 g/dL in men according to World Health Organization criteria [22].

Laboratory parameters included RDW, white blood cell (WBC), mean platelet volume (MPV), ALB, fasting blood glucose (FBG), and glycohemoglobin (GHb).

### Statistical analysis

The analysis data were weighted using the weights provided by NHANES. Continuous data were described as mean (standard error) (S.E), and differences between the two groups were compared using t-test. Counting data were described as number and percentage [n (%)], and differences between the two groups were compared using chi-squared test. Missing data were processed using multiple imputation, and sensitivity analysis was performed before and after the imputation. Spearman rank correlation coefficient was used to examine the association between RAR and the severity level of DR. Univariate and multivariate cox regression models were used to assess the association between RAR and all-cause mortality or CVD mortality, and results were shown as HR (hazard ratio) with 95% confidence intervals (CIs). Univariate cox regression model was used to screen covariates, with all-cause mortality and CVD mortality as outcomes. Model 1 was unadjusted model. Model 2 adjusted covariates with statistical significance in the univariate cox regression model. The concordance index (C-index) was used to assess the discrimination of the prediction model. Subgroup analysis was performed based on age and hyperlipidemia. Statistical analyses were performed using SAS 9.4 (SAS Institute Inc., Cary, NC, USA) and R 4.2.0 (Institute for Statistics and Mathematics, Vienna, Austria). *P* < 0.05 was considered as statistical significance.

## Results

### The selection and characteristics of patients

**Fig 1:**
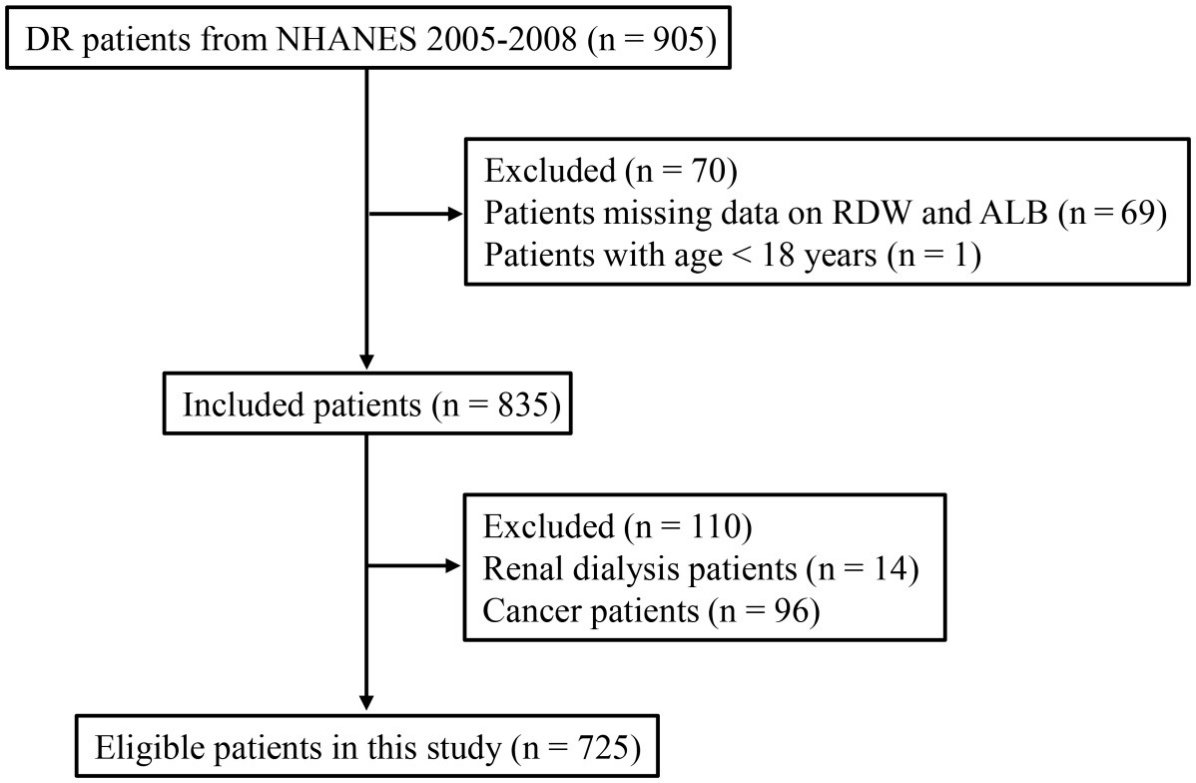
The flowchart of patient selection.

There were 905 DR patients extracted from the 2005–2008 NHANES database. After excluding 69 patients missing data on RDW and ALB and one patient with age < 18 years, 835 patients remained. Further, 14 patients with renal dialysis and 96 patients with cancer were excluded. Finally, 725 eligible patients were included in this study (Fig 1). Of these, 614 patients were classified into mild level group and 111 patients were classified into moderate/severe level group. S1 Table shows the number and percentage of missing data. The missing data were addressed by multiple imputation, and there was no significant difference before and after imputation (S1 Fig). The characteristics of included patients were shown in Table 1. There was statistical significance in race, duration of diabetes, smoking, BMI, anti-diabetic agent, CKD, anemia, WBC, MPV, ALB, FBG, GHb, RAR, and all-cause mortality between mild level group and moderate/severe level group (all *P* < 0.05).

**Table 1:** Characteristics of included patients.

Variables	Total (n = 725)	DR severity level		Statistics	*P*
		Mild (n = 614)	Moderate/severe (n = 111)		
Age, n (%)				χ^2^ = 0.022	0.881
< 65 years	445 (68.75)	374 (68.66)	71 (69.50)		
≥ 65 years	280 (31.25)	240 (31.34)	40 (30.50)		
Gender, n (%)				χ^2^ = 0.140	0.708
Male	397 (53.43)	342 (53.66)	55 (51.62)		
Female	328 (46.57)	272 (46.34)	56 (48.38)		
Race, n (%)				χ^2^ = 22.426	< 0.001
Mexican American	143 (8.02)	116 (7.39)	27 (13.09)		
Other Hispanic	57 (4.70)	50 (4.96)	7 (2.67)		
Non-Hispanic White	288 (65.13)	263 (66.87)	25 (51.26)		
Non-Hispanic Black	213 (16.02)	163 (14.24)	50 (30.30)		
Other races	24 (6.12)	22 (6.55)	2 (2.68)		
Education level, n (%)				χ^2^ = 1.760	0.780
Less than 9th grade	157 (11.78)	130 (11.43)	27 (14.63)		
9-11th grade	130 (14.08)	108 (13.62)	22 (17.74)		
High school/GED or equivalent	174 (28.71)	148 (29.09)	26 (25.69)		
Some college or AA	174 (28.09)	152 (28.51)	22 (24.77)		
College or above	90 (17.34)	76 (17.36)	14 (17.17)		
Marital status, n (%)				χ^2^ = 6.240	0.284
Married	433 (64.13)	372 (64.52)	61 (61.02)		
Widowed	102 (10.09)	82 (9.44)	20 (15.26)		
Divorced	86 (12.21)	73 (12.11)	13 (13.04)		
Separated	21 (1.99)	15 (1.81)	6 (3.45)		
Never married	56 (7.95)	49 (8.26)	7 (5.48)		
Living with partner	27 (3.63)	23 (3.86)	4 (1.75)		
PIR, ratio, Mean (S.E)	2.77 (0.10)	2.77 (0.10)	2.83 (0.21)	t = -0.33	0.747
Duration of diabetes, n (%)				χ^2^ = 12.663	0.002
< 5 years	79 (9.60)	74 (10.54)	5 (2.06)		
5–10 years	89 (12.29)	72 (11.22)	17 (20.88)		
> 10 years	557 (78.11)	468 (78.25)	89 (77.06)		
MET, MET/min, Mean (S.E)	736.11 (79.62)	753.11 (90.08)	599.99 (158.13)	t = 0.80	0.430
Smoking, n (%)				χ^2^ = 6.952	0.008
Yes	361 (48.34)	312 (50.06)	49 (34.63)		
No	364 (51.66)	302 (49.94)	62 (65.37)		
Drinking, n (%)				χ^2^ = 1.189	0.276
Yes	453 (63.14)	391 (64.06)	62 (55.72)		
No	272 (36.86)	223 (35.94)	49 (44.28)		
Family history of diabetes, n (%)				χ^2^ = 3.191	0.074
Yes	412 (54.84)	339 (53.68)	73 (64.04)		
No	313 (45.16)	275 (46.32)	38 (35.96)		
Family history of heart attack, n (%)				χ^2^ = 0.505	0.477
Yes	120 (17.64)	99 (17.08)	21 (22.13)		
No	605 (82.36)	515 (82.92)	90 (77.87)		
BMI, kg/m^2^, Mean (S.E)	30.69 (0.30)	30.30 (0.33)	33.82 (1.18)	t = -2.76	0.010
Anti-diabetic agent, n (%)				χ^2^ = 73.751	< 0.001
Yes	365 (44.13)	274 (38.98)	91 (85.30)		
No	360 (55.87)	340 (61.02)	20 (14.70)		
Glaucoma, n (%)				χ^2^ = 1.550	0.213
Yes	74 (9.02)	60 (8.54)	14 (12.85)		
No	651 (90.98)	554 (91.46)	97 (87.15)		
Macular degeneration, n (%)				χ^2^ = 2.828	0.093
Yes	31 (4.26)	24 (3.53)	7 (10.08)		
No	694 (95.74)	590 (96.47)	104 (89.92)		
Hypertension, n (%)				χ^2^ = 1.237	0.266
Yes	571 (73.55)	474 (72.70)	97 (80.40)		
No	154 (26.45)	140 (27.30)	14 (19.60)		
CVD, n (%)				χ^2^ = 1.462	0.227
Yes	192 (23.01)	153 (22.37)	39 (28.15)		
No	533 (76.99)	461 (77.63)	72 (71.85)		
Depression, n (%)				χ^2^ = 2.643	0.104
Yes	70 (8.48)	58 (7.74)	12 (14.39)		
No	655 (91.52)	556 (92.26)	99 (85.61)		
Hyperlipidemia, n (%)				χ^2^ = 0.458	0.499
Yes	618 (82.73)	518 (82.33)	100 (85.93)		
No	107 (17.27)	96 (17.67)	11 (14.07)		
CKD, n (%)				χ^2^ = 13.879	< 0.001
Yes	159 (18.04)	121 (16.04)	38 (34.02)		
No	566 (81.96)	493 (83.96)	73 (65.98)		
CLD, n (%)				χ^2^ = 0.206	0.650
Yes	36 (6.63)	34 (6.84)	2 (4.93)		
No	689 (93.37)	580 (93.16)	109 (95.07)		
Anemia, n (%)				χ^2^ = 14.394	< 0.001
No	569 (81.69)	499 (83.44)	70 (67.73)		
Yes	156 (18.31)	115 (16.56)	41 (32.27)		
RDW, %, Mean (S.E)	13.04 (0.08)	13.02 (0.08)	13.18 (0.12)	t = -1.25	0.220
WBC, 1000 cells/uL, Mean (S.E)	7.38 (0.13)	7.23 (0.11)	8.57 (0.65)	t = -2.07	0.047
MPV, fL, Mean (S.E)	7.98 (0.05)	7.95 (0.05)	8.23 (0.10)	t = -2.65	0.012
ALB, g/L, Mean (S.E)	41.41 (0.18)	41.57 (0.21)	40.11 (0.33)	t = 3.37	0.002
FBG, mmol/L, Mean (S.E)	7.54 (0.17)	7.25 (0.17)	9.89 (0.81)	t = -3.09	0.004
GHb, %, Mean (S.E)	6.66 (0.07)	6.47 (0.07)	8.17 (0.29)	t = -5.51	< 0.001
RAR, Mean (S.E)	0.32 (0.00)	0.32 (0.00)	0.33 (0.00)	t = -2.96	0.006
All-cause mortality, n (%)				χ^2^ = 4.192	0.041
Yes	264 (32.41)	210 (30.85)	54 (44.87)		
No	461 (67.59)	404 (69.15)	57 (55.13)		
CVD-cause mortality, n (%)				χ^2^ = 1.120	0.290
Yes	94 (11.83)	73 (11.38)	21 (15.41)		
No	631 (88.17)	541 (88.62)	90 (84.59)		
Follow-up					
Follow up time, month, Mean (S.E)	133.84 (2.85)	134.78 (3.15)	126.32 (5.63)	t = 1.33	0.193

Abbreviation: DR, diabetic retinopathy; GED, General Educational Development; AA, associate of arts; PIR, poverty income ratio; MET, metablic equivalent; CVD, cardiovascular diseases; CKD, chronic kidney disease; CLD, chronic liver disease; BMI, body mass index; RDW, red blood cell distribution width; WBC, white blood cell; MPV, mean platelet volume; ALB, albumin; FBG, fasting blood glucose; GHb, glycohemoglobin; RAR, red blood cell distribution width/albumin; mean (S.E), mean (standard error).

### Association between RAR and the severity level of DR

The association between RAR and severity level of DR was shown in Table 2. The result displayed that RAR was positively associated with the severity level of DR (*P* < 0.001).

**Table 2:** The association between RAR and DR severity.

Variables	DR severity	
	Correlation	*P*
RAR	0.169	< 0.001

Abbreviation: RAR, red blood cell distribution width/albumin; DR, diabetic retinopathy.

Regarding all-cause mortality as the outcome, age, race, education level, marital status, PIR, MET, smoking, drinking, anti-diabetic agent, macular degeneration, hypertension, CVD, CKD, WBC, FBG, and GHb were identified as covariates. Regarding CVD mortality as the outcome, age, race, marital status, PIR, family history of heart attack, anti-diabetic agent, hypertension, CVD, hyperlipidemia, CKD, and MPV were identified as covariates. The results were shown in S2 Table. Further, we adjusted the severity level of DR.

Table 3 shows the association between RAR and the risk of all-cause mortality and CVD mortality. In the unadjusted model, we found that higher RAR was associated with the higher risk of all-cause mortality and CVD mortality, respectively (both *P* < 0.05). After adjusting the above covariates, we found that the increase of RAR was associated with the increased risk of all-cause mortality (HR: 1.15, 95%CI: 1.01–1.31) and CVD mortality (HR: 1.35, 95%CI: 1.12–1.63), respectively.

**Table 3:** Association between RAR and all-cause mortality or CVD mortality.

Model	All-cause mortality*		CVD mortality^#^	
	HR (95%CI)	*P*	HR (95%CI)	*P*
Model 1	1.33 (1.20–1.47)	< 0.001	1.38 (1.21–1.59)	< 0.001
Model 2	1.15 (1.01–1.31)	0.039	1.35 (1.12–1.63)	0.002

Abbreviation: HR, hazard ratio; CI, confidence interval; RAR, red blood cell distribution width/albumin; CVD, cardiovascular diseases.

Model 1 Unadjusted model

Model 2* Adjusted age, race, education level, marital status, PIR, MET, smoking, drinking, anti-diabetic agent, macular degeneration, hypertension, CVD, CKD, WBC, FBG, GHb, and DR severity level.

Model 2^#^ Adjusted age, race, marital status, PIR, family history of heart attack, anti-diabetic agent, hypertension, CVD, hyperlipidemia, CKD, MPV, and DR severity level.

### Predictive value of RAR for all-cause mortality and CVD mortality in DR patients

We also examined the predictive ability of RAR in the all-cause mortality and CVD mortality. The results showed that the C-index of RAR for all-cause mortality and CVD mortality was 0.63 (95%CI: 0.59–0.67) and 0.65 (95%CI: 0.59–0.71), respectively (Table 4).

**Table 4:** Predictive value of RAR in all-cause mortality and CVD mortality.

Variables	C-index (95%CI)
All-cause mortality	0.63 (0.59–0.67)
CVD mortality	0.65 (0.59–0.71)

Abbreviation: C-index, concordance index; CI, confidence interval; RAR, red blood cell distribution width/albumin; CVD, cardiovascular diseases.

### Association between RAR and the prognosis of DR based on age and hyperlipidemia

In age subgroup, the increase of RAR was associated with the higher risk of CVD mortality (HR: 1.35, 95%CI: 1.09–1.67) while not associated with the risk of all-cause mortality (HR: 1.12, 95%CI: 0.96–1.31) in DR patients with age < 65 years. There was no statistical significance between RAR and all-cause mortality or CVD mortality in patients with age ≥ 65 years (both *P* > 0.05). In hyperlipidemia subgroup, the increase of RAR was associated with the higher risk of CVD mortality (HR: 1.34, 95%CI: 1.10–1.64), and no statistical significance was found in the risk of all-cause mortality (HR: 1.11, 95%CI: 0.93–1.33) in DR patients with hyperlipidemia. There was no significant difference in the risk of all-cause mortality in DR patients without hyperlipidemia (HR: 1.57, 95%CI: 0.88–2.83) (Table 5).

**Table 5:** Association between RAR and all-cause mortality or CVD mortality in DR patients with different ages or hyperlipidemia (or not).

Model	All-cause mortality*		CVD mortality^#^	
	HR (95%CI)	*P*	HR (95%CI)	*P*
Age				
< 65 years	1.12 (0.96–1.31)	0.152	1.35 (1.09–1.67)	0.006
≥ 65 years	0.98 (0.74–1.29)	0.870	0.83 (0.54–1.30)	0.418
Hyperlipidemia				
Yes	1.11 (0.93–1.33)	0.238	1.34 (1.10–1.64)	0.003
No	1.57 (0.88–2.83)	0.129	-	-

Abbreviation: RAR, red blood cell distribution width/albumin; CVD, cardiovascular diseases; DR, diabetic retinopathy; HR, hazard ratio; CI, confidence interval.

* Adjusted age (or not), race, education level, marital status, PIR, MET, smoking, drinking, anti-diabetic agent, macular degeneration, hypertension, CVD, CKD, WBC, FBG, GHb, and DR severity level.

^#^ Adjusted age (or not), race, marital status, PIR, family history of heart attack, anti-diabetic agent, hypertension, CVD, hyperlipidemia (or not), CKD, MPV, and DR severity level.

## Discussion

The all-cause mortality and CVD mortality show an increasing trend in DR patients [1, 3]. Thus, it is important to explore some valuable indicators to improve the prognosis of DR patients. RAR is a combined indicator, and has been reported as an independent risk factor for DR [15]. A study has reported that RAR was a robust and independent marker to predict the all-cause mortality in patients with diabetic foot ulcers [7]. Herein, we explored the association and prediction ability of RAR in the prognosis of DR patients. The results showed that increase of RAR was associated with the increased risk of all-cause mortality and CVD mortality. We also found the positive association between RAR and CVD mortality in DR patients with age < 65 years or hyperlipidemia. In addition, RAR was a useful indicator to predict the prognosis of DR patients.

RDW increases with inflammatory stimulation [23], and increase of RDW is related to the adverse outcomes in several inflammation-related diseases [24–26]. Previous studies have reported the association between RAR and disease development or mortality in patients with diabetes-related complications, such as diabetic nephropathy [27], diabetic foot ulcers [7], and diabetic ketoacidosis [28]. Retinal hypoxia and ischaemia are the main reasons for neovascularization and vascular dystrophies in DR patients [4]. Evidence has shown the increase of RDW in other ocular diseases accompanied by retinal hypoxia [29–31]. The mechanisms for the increase of RDW in DR patients remain unclear. One potential explaining is that DR is accompanied by the inflammatory response, hypoxia, and abnormal vascular endothelial function [32], which impairs the generation and/or abnormal survival of red blood cell, thereby leading to the increase of RDW [33]. ALB is also an inflammation-related indicator [34]. In inflammatory states, capillary permeability and the escape of ALB are increased, which expands interstitial space and increases the distribution volume of ALB [34]. In addition, the half-life of ALB is shown to be shortened, which decreases the total mass of ALB [34]. These two factors lead to hypoalbuminemia. Therefore, hypoalbuminemia reflects the inflammatory state, and is associated with the shortened life span [34]. Low ALB concentration is correlated with the higher risk of mortality in patients with diabetic ketoacidosis [35]. Existing evidence has shown the more accuracy of combined inflammation-related indexes to predict the mortality [36, 37]. Previous studies have shown that RAR was positively associated with the all-cause mortality of patients with diabetic complications [7, 38]. Consistent with the results from previous studies, we found that increase of RAR was associated with the increased risk of all-cause mortality in DR patients. Also, the risk of CVD mortality was increased with the increase of RAR in DR patients.

Age has been reported to be associated with DR, and DR is the main reason for blindness in people at working age [39]. Hong et al. have found that RAR was an effective predictor for the all-cause mortality in patients with diabetic foot ulcers at age < 65 years [7]. In this study, although there was no significant difference, we found that higher RAR was associated with the higher risk of all-cause mortality in DR patients at age < 65 years. Moreover, higher RAR was found to be significantly associated with the higher risk of CVD mortality. These findings suggested that DR patients with age < 65 years should pay more attention to the increase of RAR. CVD is the leading cause of mortality in adults, and compared to people with normal total cholesterol levels, hyperlipidemia patients are at roughly twice the risk of developing CVD [40]. Our study found that DR patients with hyperlipidemia had a higher risk of CVD mortality with the increase of RAR, which indicated the importance of decreasing RAR level in DR patients with hyperlipidemia.

Our study also found the good predictive performance of RAR in the risk of all-cause mortality and CVD mortality. Previous studies have reported that RDW and ALB could independently predict the prognosis of patients with diabetes-related complications [7, 41]. RAR was easily calculated in clinical practice, and showed a good performance to predict the all-cause mortality [37]. Our finding displayed that RAR may be a simple and effective predictor for the prognosis of DR patients.

We explore the association between RAR and mortality risk of DR patients and prove the predictive value of RAR in the prognosis. This is a cohort study, which can reveal the causal association. Moreover, the indexes (RDW and ALB) used in this study are simple and easy to measure, which are convenient to apply. However, there are some limitations in this study. First, the sample size in our study may be relatively small. However, the sample is extracted from the NHANES database. This database includes a nationally representative sample and uses a complex, multistage, probability sampling design, which makes up for this limitation to a certain extent. Second, this is a retrospective study, and recall bias or incorrect records is inevitable. However, DR patients included in this study are definitely diagnosed by retinal image, which decreases the bias. Third, changes in treatments and lifestyles may affect the results. Due to the defects of database, we cannot further analyze based on treatments and lifestyles. In the future, more studies are needed to verify our findings.

## Conclusion

In conclusion, we found that higher RAR was associated with the higher risk of all-cause mortality and CVD mortality in DR patients. RAR was a useful predictor for the prognosis of DR patients. Our findings suggested that clinicians may use RAR to predict the risk of all-cause mortality and CVD mortality for DR patients, which may help them take appropriate treatments to improve the prognosis of DR patients at high-risk.

## Supporting information

S1 Fig Sensitivity analysis for missing data before and after imputation. (TIF)

S1 Table The number and percentage of missing data. (DOCX)

S2 Table The selection of covariates. (DOCX)
