# Supplementary figures and images for: Association between red blood cell distribution width/albumin ratio and all-cause mortality or cardiovascular diseases mortality in patients with diabetic retinopathy: A cohort study
Source: PLoS One. 2023 Dec 21;18(12):e0296019. doi: 10.1371/journal.pone.0296019 (PMC10735013; doi:10.1371/journal.pone.0296019)

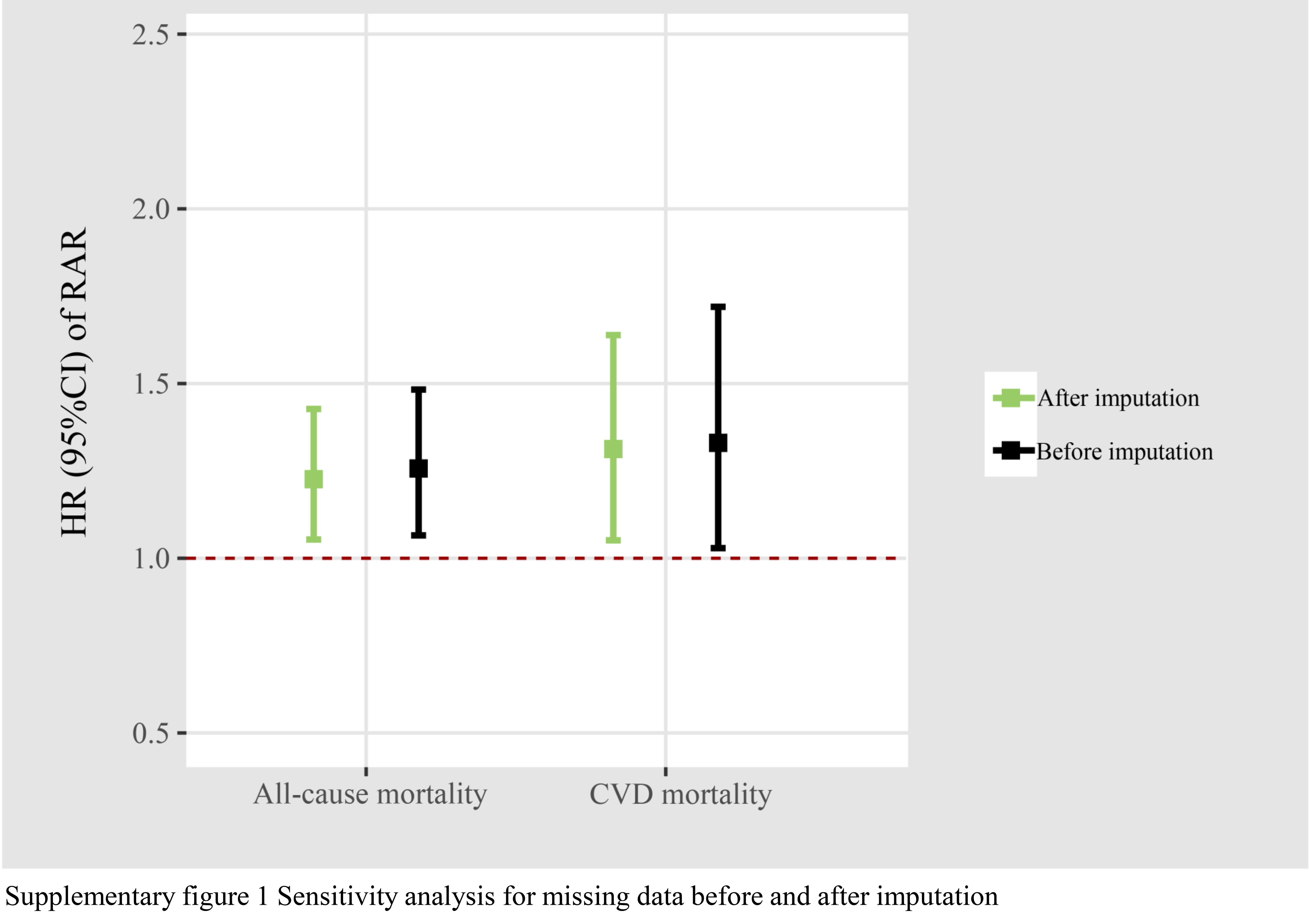

Supplement: S1 Fig — (TIF) [file pone.0296019.s001.tif]
