# Supplementary material for: Association between red blood cell distribution width/albumin ratio and all-cause mortality or cardiovascular diseases mortality in patients with diabetic retinopathy: A cohort study
Source: PLoS One. 2023 Dec 21;18(12):e0296019. doi: 10.1371/journal.pone.0296019 (PMC10735013; doi:10.1371/journal.pone.0296019)
Supplement: S1 Table — (DOCX) [file pone.0296019.s002.docx]

**S1 Table The number and percentage of missing data**

| **Variables** | **Missing [n (%)]** |
| --- | --- |
| Education level | 1 (0.14) |
| Marital status | 1 (0.14) |
| PIR | 55 (7.59) |
| Smoking | 1 (0.14) |
| Drinking | 29 (4.00) |
| Diabetes family history | 1 (0.14) |
| Heart attack family history | 1 (0.14) |
| Glaucoma | 29 (4.00) |
| Macular degeneration | 31 (4.28) |
| BMI | 14 (1.93) |
| GHb | 5 (0.69) |
| CVD | 9 (1.24) |

Abbreviation: PIR, ratio of family income to poverty; BMI, body mass index; GHb, glycohemoglobin; CVD, cardiovascular diseases.
