# Supplementary material for: Association between red blood cell distribution width/albumin ratio and all-cause mortality or cardiovascular diseases mortality in patients with diabetic retinopathy: A cohort study
Source: PLoS One. 2023 Dec 21;18(12):e0296019. doi: 10.1371/journal.pone.0296019 (PMC10735013; doi:10.1371/journal.pone.0296019)
Supplement: S2 Table — (DOCX) [file pone.0296019.s003.docx]

**S2 Table The selection of covariates**

| **Variables** | **All-cause mortality** | | **CVD mortality** | |
| --- | --- | --- | --- | --- |
|  | **HR (95%CI)** | ***P*** | **HR (95%CI)** | ***P*** |
| Age | 1.08 (1.07-1.09) | < 0.001 | 1.10 (1.07-1.12) | < 0.001 |
| Gender |  |  |  |  |
| Male | Ref |  | Ref |  |
| Female | 1.11 (0.87-1.42) | 0.416 | 1.23 (0.81-1.89) | 0.332 |
| Race |  |  |  |  |
| Mexican American | Ref |  | Ref |  |
| Other Hispanic | 0.17 (0.07-0.42) | < 0.001 | 0.24 (0.05-1.14) | 0.072 |
| Non-Hispanic White | 1.52 (0.92-2.52) | 0.101 | 2.98 (1.13-7.84) | 0.027 |
| Non-Hispanic Black | 1.19 (0.67-2.11) | 0.549 | 2.02 (0.69-5.88) | 0.198 |
| Other races | 0.95 (0.34-2.63) | 0.919 | 0.00 (0.00-0.00) | < 0.001 |
| Education level |  |  |  |  |
| Less than 9th grade | Ref |  | Ref |  |
| 9-11th grade | 0.87 (0.55-1.38) | 0.544 | 1.13 (0.53-2.40) | 0.752 |
| High school /GED or equivalent | 0.76 (0.49-1.17) | 0.216 | 0.80 (0.32-1.96) | 0.620 |
| Some college or AA degree | 0.64 (0.38-1.08) | 0.098 | 0.95 (0.38-2.38) | 0.916 |
| College graduate or above | 0.27 (0.16-0.47) | < 0.001 | 0.44 (0.15-1.28) | 0.129 |
| Marital status |  |  |  |  |
| Married | Ref |  | Ref |  |
| Widowed | 3.56 (2.51-5.04) | < 0.001 | 6.23 (3.58-10.84) | < 0.001 |
| Divorced | 1.01 (0.57-1.79) | 0.970 | 0.59 (0.24-1.43) | 0.240 |
| Separated | 1.25 (0.73-2.14) | 0.425 | 1.23 (0.29-5.22) | 0.781 |
| Never married | 0.86 (0.44-1.69) | 0.669 | 1.72 (0.69-4.33) | 0.247 |
| Living with partner | 1.17 (0.55-2.47) | 0.687 | 2.51 (0.97-6.48) | 0.057 |
| PIR | 0.66 (0.55-0.78) | < 0.001 | 0.78 (0.63-0.98) | 0.036 |
| Duration of diabetes |  |  |  |  |
| < 5 years | Ref |  | Ref |  |
| 5-10 years | 1.20 (0.76-1.88) | 0.428 | 1.25 (0.61-2.57) | 0.548 |
| > 10 years | 1.11 (0.77-1.59) | 0.590 | 0.97 (0.47-2.01) | 0.938 |
| MET | 0.69 (0.52-0.92) | 0.013 | 0.72 (0.48-1.09) | 0.119 |
| Smoking |  |  |  |  |
| No | Ref |  | Ref |  |
| Yes | 1.45 (1.08-1.95) | 0.013 | 1.53 (0.90-2.60) | 0.118 |
| Drinking |  |  |  |  |
| No | Ref |  | Ref |  |
| Yes | 0.67 (0.48-0.94) | 0.021 | 0.83 (0.54-1.28) | 0.401 |
| Family history of diabetes |  |  |  |  |
| No | Ref |  | Ref |  |
| Yes | 1.06 (0.77-1.47) | 0.711 | 1.02 (0.68-1.51) | 0.938 |
| Family history of heart attack |  |  |  |  |
| No | Ref |  | Ref |  |
| Yes | 1.23 (0.96-1.58) | 0.097 | 1.73 (1.12-2.68) | 0.013 |
| BMI | 1.15 (0.98-1.34) | 0.085 | 1.01 (0.83-1.23) | 0.919 |
| Anti-diabetic agent |  |  |  |  |
| No | Ref |  | Ref |  |
| Yes | 2.11 (1.54-2.88) | < 0.001 | 1.93 (1.10-3.37) | 0.021 |
| Glaucoma |  |  |  |  |
| No | Ref |  | Ref |  |
| Yes | 1.36 (0.86-2.17) | 0.191 | 1.47 (0.51-4.28) | 0.479 |
| Macular degeneration |  |  |  |  |
| No | Ref |  | Ref |  |
| Yes | 2.13 (1.16-3.93) | 0.015 | 2.07 (0.80-5.35) | 0.131 |
| Hypertension |  |  |  |  |
| No | Ref |  | Ref |  |
| Yes | 2.99 (1.97-4.53) | < 0.001 | 3.11 (1.35-7.17) | 0.008 |
| CVD |  |  |  |  |
| No | Ref |  | Ref |  |
| Yes | 2.79 (2.09-3.73) | < 0.001 | 3.23 (1.93-5.41) | < 0.001 |
| Depression |  |  |  |  |
| No | Ref |  | Ref |  |
| Yes | 1.19 (0.75-1.87) | 0.458 | 0.97 (0.40-2.33) | 0.940 |
| Hyperlipidemia |  |  |  |  |
| No | Ref |  | Ref |  |
| Yes | 1.11 (0.71-1.74) | 0.634 | 1.98 (1.07-3.68) | 0.030 |
| CKD |  |  |  |  |
| No | Ref |  | Ref |  |
| Yes | 3.92 (2.68-5.72) | < 0.001 | 3.69 (1.93-7.02) | < 0.001 |
| CLD |  |  |  |  |
| No | Ref |  | Ref |  |
| Yes | 0.92 (0.43-1.96) | 0.833 | 1.05 (0.36-3.04) | 0.924 |
| Anemia |  |  |  |  |
| No | Ref |  | Ref |  |
| Yes | 1.24 (0.97-1.60) | 0.090 | 1.34 (0.88-2.04) | 0.166 |
| WBC | 1.20 (1.07-1.34) | 0.001 | 1.12 (0.94-1.33) | 0.222 |
| MPV | 1.15 (0.99-1.35) | 0.067 | 1.31 (1.04-1.65) | 0.019 |
| FBG | 1.16 (1.04-1.29) | 0.008 | 0.97 (0.81-1.16) | 0.737 |
| GHb | 1.17 (1.04-1.32) | 0.011 | 0.92 (0.74-1.14) | 0.452 |

Abbreviation: HR, hazard ratio; CI, confidence interval; CVD, cardiovascular diseases; GED, General Educational Development; AA, associate of arts; PIR, ratio of family income to poverty; MET, metablic equivalent; BMI, body mass index; WBC, white blood cell; MPV, mean platelet volume; FBG, fasting blood glucose; GHb, glycohemoglobin.
